# New Insectotoxin from Tibellus Oblongus Spider Venom Presents Novel Adaptation of ICK Fold

**DOI:** 10.3390/toxins13010029

**Published:** 2021-01-04

**Authors:** Yuliya Korolkova, Ekaterina Maleeva, Alexander Mikov, Anna Lobas, Elizaveta Solovyeva, Mikhail Gorshkov, Yaroslav Andreev, Steve Peigneur, Jan Tytgat, Fedor Kornilov, Vladislav Lushpa, Konstantin Mineev, Sergey Kozlov

**Affiliations:** 1 Department of Molecular Neurobiology, Shemyakin-Ovchinnikov Institute of Bioorganic Chemistry RAS, 16/10 Miklukho-Maklay Str., 117997 Moscow, Russia; katerina@1ns.ru (E.M.); mikov.alexander@gmail.com (A.M.); ay@land.ru (Y.A.); serg@ibch.ru (S.K.); 2 Skolkovo Institute of Science and Technology, 30 Bld. 1, Bolshoy Boulevard, 121205 Moscow, Russia; 3 V.L. Talrose Institute for Energy Problems of Chemical Physics, N.N. Semenov Federal Research Center for Chemical Physics, Russian Academy of Sciences, 38 Leninsky Pr., Bld. 2, 119334 Moscow, Russia; lobas.anka@gmail.com (A.L.); lisavetasol@gmail.com (E.S.); mike.gorshkov@gmail.com (M.G.); 4 Department of Molecular and Chemical Physics, Moscow Institute of Physics and Technology (National Research University), 9 Institutsky Per., 141700 Dolgoprudny, Russia; 5 Moscow Institute of Molecular Medicine, Sechenov First Moscow State Medical University, 8 Bld. 2, Trubetskaya Str., 119991 Moscow, Russia; 6 Toxicology and Pharmacology, University of Leuven (KU Leuven), Campus Gasthuisberg, O&N2, Herestraat 49, P.O. Box 922, B-3000 Leuven, Belgium; steve.peigneur@kuleuven.be (S.P.); jan.tytgat@kuleuven.be (J.T.); 7 Department of Biological and Medicinal Physics, Moscow Institute of Physics and Technology (National Research University), 9 Institutsky Per., 141700 Dolgoprudny, Russia; kornilov.fd@phystech.edu (F.K.); lushpa@phystech.edu (V.L.); konstantin.mineev@gmail.com (K.M.); 8 Department of Structural Biology, Shemyakin–Ovchinnikov Institute of Bioorganic Chemistry RAS, 16/10 Miklukho-Maklay Str., 117997 Moscow, Russia

**Keywords:** spider venom, transcriptome, proteome, insectotoxin, ICK fold, NMR structure

## Abstract

The *Tibellus oblongus* spider is an active predator that does not spin webs and remains poorly investigated in terms of venom composition. Here, we present a new toxin, named Tbo-IT2, predicted by cDNA analysis of venom glands transcriptome. The presence of Tbo-IT2 in the venom was confirmed by proteomic analyses using the LC-MS and MS/MS techniques. The distinctive features of Tbo-IT2 are the low similarity of primary structure with known animal toxins and the unusual motif of 10 cysteine residues distribution. Recombinant Tbo-IT2 (rTbo-IT2), produced in *E. coli* using the thioredoxin fusion protein strategy, was structurally and functionally studied. rTbo-IT2 showed insecticidal activity on larvae of the housefly *Musca domestica* (LD_100_ 200 μg/g) and no activity on the panel of expressed neuronal receptors and ion channels. The spatial structure of the peptide was determined in a water solution by NMR spectroscopy. The Tbo-IT2 structure is a new example of evolutionary adaptation of a well-known inhibitor cystine knot (ICK) fold to 5 disulfide bonds configuration, which determines additional conformational stability and gives opportunities for insectotoxicity and probably some other interesting features.

## 1. Introduction

Spiders are the second-largest taxonomic group of terrestrial organisms after insects. A huge variety of spider species (about 48’907 species according to World Spider Catalog (2020) (http://www.wsc.nmbe.ch) has actually no analogs in the living world among venomous animals. It has been shown that each species of spider has at least 100 unique peptide toxins in its venom [1]. Consequently, the total number of individual spider toxins seems to be quite amazing. However, all this potential diversity of biologically active peptides remains poorly investigated. ArachnoServer (http://www.arachnoserver.org) reports about 1576 spider toxins (on November 2020) from 100 different spider species studied to date, but less than 490 toxins have a described target. It is a tiny part of all spider venom toxins, among which, as believed, molecules that act on almost all molecular targets can be found.

Spider venom is composed of proteins, including enzymes [1,2] and large toxins (e.g., α-latrotoxin from black widow spider venom, a powerful presynaptic secretagogue [3]), small peptides, and low molecular weight compounds [1]. Small peptides usually make up the majority of the venom and can be divided into linear (not containing intramolecular disulfide bonds) and disulfide-stabilized. Linear peptides were found only in some spider families and possessed cytolytic (antimicrobial) activity. Cysteine-rich spider peptides, among which the most diverse are knottins (with inhibitor cystine knot (ICK) structural fold), are the most interesting since they have a rigidly stabilized spatial structure and exhibit neurotoxic properties. The targets of these peptides are ion channels, such as voltage-gated sodium channels [4,5], potassium channels [6,7], calcium channels [8,9], acid-sensing ion channels [10], transient receptor potential (TRP) [11,12], and purinergic ion channels (P2X) [13,14]. Natural molecules from spider venoms are considered as potential therapeutics against pathophysiological conditions including cancer and pain, and for the development of novel bioinsecticides for agricultural use [15,16].

*Tibellus oblongus* (Walckenaer, 1802) is distributed throughout the Holarctic ecozone and belongs to the Philodromidae family consisting of 539 species. *T. oblongus*, as well as all other species of the *Tibellus* genus, is an active predator that does not spin webs, but actively pursues its prey. Earlier, we described *T. oblongus* insectotoxin ω-Tbo-IT1 [17]—the only studied toxin from the entire Philodromidae family to date. Here, we present the second toxin found in the diversity of *T. oblongus* spider toxins using transcriptome and proteome analysis methods. We report recombinant toxin production and 3D structure determination of a novel insecticidal 10-Cys polypeptide—rTbo-IT2, which adopted ICK fold with 5 disulfide bridges.

## 2. Results

### 2.1. Tbo-IT2 Primary Structure

#### 2.1.1. Transcriptome Analysis of cDNA Sequencing Data from the Venom Glands of *Tibellus Oblongus*

Total RNA was extracted from the glands of the spider *T. oblongus* using the SV Total RNA Isolation System kit. The cDNA library was obtained by PCR using the SMART cDNA Library Construction Kit using the standard methodology recommended by the manufacturer. As a result of automatic Sanger sequencing of cloned cDNA, a database of expressed sequence tags (EST) corresponding to the peptides encoded in the mRNA was obtained. The resulting transcripts library consisted of 1733 clones (unpublished data, a matter of a separate publication), each of which has been analyzed as a separate independent transcript for all possible translation variants. About 1200 different precursor proteins were deduced, among which more than 800 sequences were unique. The group of cysteine-containing toxins included 217 different deduced sequences that have been grouped into homologous families.

#### 2.1.2. Tbo-IT2 Precursor Determination

**Figure 1:**
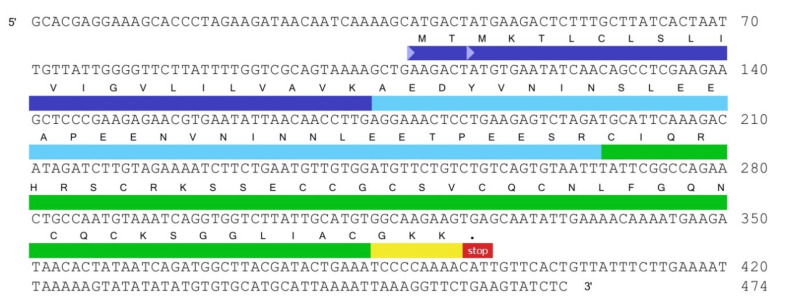
Nucleotide sequence of one of the expressed sequence tags (EST) database clones, encoding the Tbo-IT2 peptide precursor. Navy blue highlights the signal peptide region, cyan—the propeptide fragment, green—the mature peptide, yellow—the post-translational modification.

Among cysteine-containing peptides, one sequence with an uncommon cysteine residues distribution pattern did not belong to any homologous family and, therefore, was selected for further investigation. Seventeen separate clones encoding the Tbo-IT2 precursor peptide in one of the translation frames were found in the database of EST fragments. The precursor protein consisted of a signal peptide, a propeptide fragment, and 42 residue-length mature sequence followed by a stop codon (Figure 1). The pre-propeptide structure was in good accordance with spider toxin maturation principles [18,19]. Cleavage of three C-terminal residues with the formation of an amide is possible as a post-translational modification [19].

### 2.2. Identification of Tbo-IT2 in the Spider Venom

#### 2.2.1. Proteomic Experiments Overview

The transcriptomic analysis of the venom gave an overview of its potential molecular constitution. To understand which toxins are actually translated as polypeptides, a proteomics analysis was performed. We used the most widespread bottom-up approach for the proteomic analysis of protein (polypeptide) mixtures, based on the specific digest of polypeptide chains and subsequent chromatography-mass spectrometric (LC-MS) analysis of the obtained proteolytic peptides. In the present study, two digests, using trypsin and GluC endoproteinase, were performed and analyzed in order to achieve higher sequence coverage and identification confidence. The main source of information about the sequence in such experiment is fragmentation mass spectra (MS/MS), and data analysis is performed using a search engine (we used IdentiPy [20]), followed by validation and analysis of the obtained identifications using in-house Python scripts based on Pyteomics library [21].

As a source of complementary information about polypeptide components of the venom, an LC-MS analysis was performed on intact species without proteolytic digestion. In the obtained data, the presence of the polypeptide of interest was confirmed by detection of the corresponding chromatographic elution peak and isotopic clusters with detection of its monoisotopic mass.

The database used for peptide matching included all propeptide and mature peptide sequences corresponding to 217 toxins predicted from transcriptome analysis, giving a total of 434 target sequences. The equal number of reversed sequences were added to the fasta file as decoys.

#### 2.2.2. Proteomic Identification of Tbo-IT2

**Figure 2:**
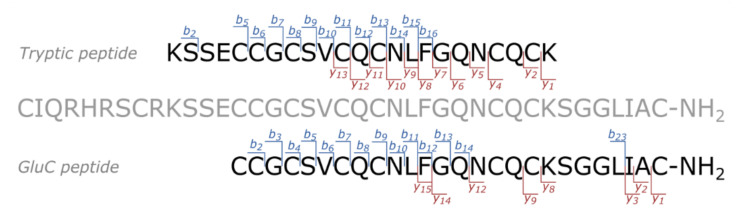
The amino acid sequence of Tbo-IT2 toxin (shown in gray) and the corresponding proteolytic peptides (shown in black) detected in tryptic (top) and GluC (bottom) digests. The *b*- and *y*-fragments detected in the MS/MS spectra corresponding to the best PSMs for each peptide are shown in blue and red, respectively.

For Tbo-IT2, two unique tryptic peptides (differing in a single lysine residue due to a missed cleavage) and one unique GluC peptide were reliably identified based on the searches of bottom-up LC-MS/MS data with a significant number of peptide-spectrum matches (PSMs) per peptide (Table 1). The GluC peptide covered the C-terminus of the toxin, therefore, confirming its amidation. Figure 2 shows two proteolytic peptides from different proteases and their coverage by *b*- and *y*-ions achieved in the corresponding MS/MS spectra.

**Figure 3:**
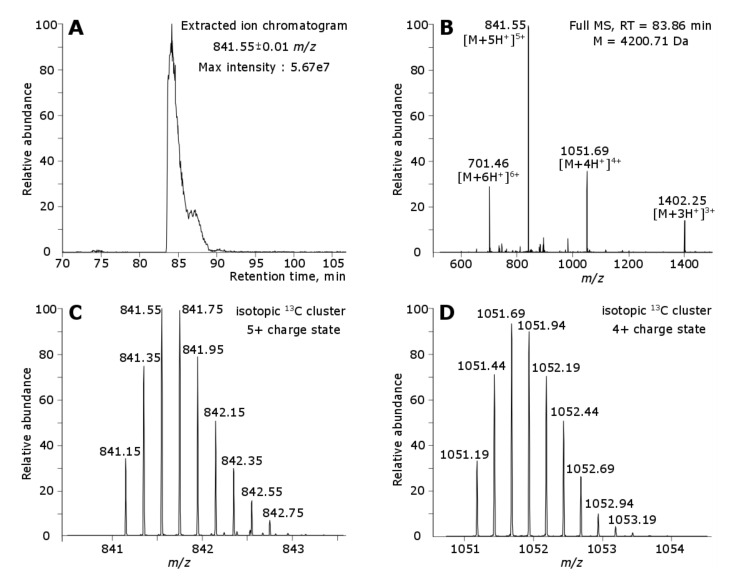
LC-MS/MS analysis of intact toxins from *Tibellus oblongus* spider venom. The intact Tbo-IT2 toxin was detected with 5 disulfide bonds and C-terminal amidation. (**A**) The extracted ion chromatogram of the most abundant isotope of Tbo-IT2 protein (841.55 m/z). (**B**) The mass-spectra of the intact Tbo-IT2 in four different charge states. The mass spectra of isotopic 13C cluster of intact Tbo-IT2 in (**C**) 5+ charge state and (**D**) 4+ charge state.

Manual search for the isotope clusters corresponding to the intact toxin in LC-MS analysis was performed, and it was shown that Tbo-IT2 polypeptide is indeed present in the tested venom (Figure 3), with a regular LC elution peak shown by extracted ion chromatogram, multiple charge states, and the monoisotopic mass (4200.71 Da), equal to theoretically calculated one with five disulfide bonds and a C-terminal amide. No proteolytic peptides corresponding to unique parts of Tbo-IT2 propeptide sequence were identified by the bottom-up approach. The intact propeptide was not detected in the LC-MS analysis.

### 2.3. Production of Recombinant Peptide

The gene encoding Tbo-IT2 was constructed by PCR considering the codons optimal for *Escherichia coli* and cloned into expression vector pET32b (+) intended for the production of recombinant peptides as a fusion protein with thioredoxin, which contributes to the correct folding of cysteine-rich molecules [22]. The expression construct was created accounting for the possible method of subsequent separation of the peptide from thioredoxin: as Tbo-IT2 does not contain methionine residues in the sequence, a methionine residue was introduced in position (−1) for hydrolysis of the fusion protein by CNBr, which we previously successfully used to obtain complex cysteine-rich peptides [23]. Expression was performed in *Escherichia coli* BL21(DE3) cells. The fusion protein was isolated by metal affinity chromatography and cleaved by CNBr to release the recombinant peptide. The recombinant peptide was purified by reversed-phase high-performance liquid chromatography (HPLC) (Figure S1 in Supplementary Materials). No additional refolding was required. The average yield of the target peptide was about 3.4 mg/liter of the cell culture. The molecular weight of the recombinant product was equal to the natural one but with a difference due to the absence of C-terminal amidation (Figure S2 in Supplementary Materials).

### 2.4. Study of the Biological Activity of Recombinant Tbo-IT2

#### 2.4.1. Insectotoxicity

**Figure 4:**
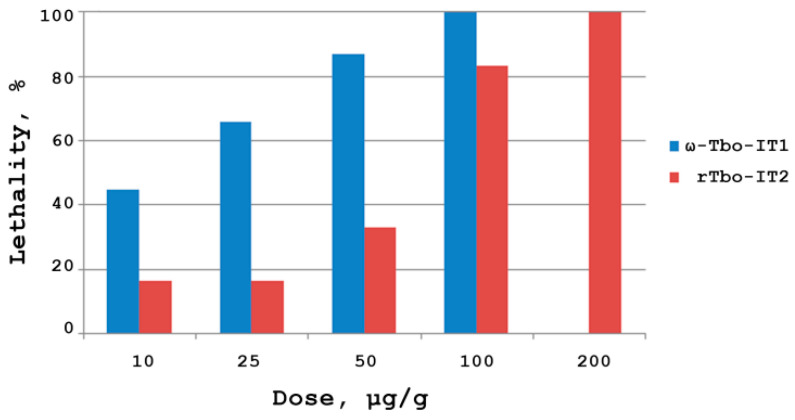
Dose-dependent insectotoxicity of rTbo-IT2 in comparison with ω-Tbo-IT1. The measurement was performed on a group of 12 house fly larvae for each dose (10, 25, 50, 100, 200 μg/g).

Insectotoxicity of the recombinant peptide Tbo-IT2 (rTbo-IT2) was tested on larvae of the housefly Musca domestica weighing about 50 mg. Lyophilized peptide samples were dissolved in pure water, and fixed aliquots were injected into the bodies of the larvae using a 1 μL syringe (Hamilton, USA). Preliminary testing was carried out at doses of 1 and 100 μg/g. Toxin rTbo-IT2 showed significant insectoactivity, with a concentration-dependent effect of action (Figure 4). ω-Tbo-IT1 was used as a comparison sample. The development of the paralyzing effect was monitored for 48 h, each dose was measured on a group of 12 larvae. The measured lethal dose LD_100_ was 200 μg/g.

#### 2.4.2. Action on Neuronal Receptors

Recombinant Tbo-IT2 was tested for TRP-receptors (rTRPV1, mTRPV2, hTRPV3, rTRPA1), stably expressed in CHO cells, using the method of calcium imaging. At a concentration of up to 1 μM, no activity was detected.

The activity of the recombinant peptide on a number of ion channels expressed in oocytes of the African clawed frog *Xenopus laevis* was also tested by electrophysiological methods. At the concentration of 5 μM, rTbo-IT2 did not show any activity toward sodium channels of insects (BgNav, DmNav, VdNav) and mammals (Nav1.4, Nav1.6, Nav1.7), and also did not act on potassium channels (Shaker, Kv1.1, Kv1.2, Kv1.3, Kv1.4, Kv1.5, Kv1.6, Kv2.1, Kv3.1, Kv4.3, Kv10.1) and calcium channel (Cav3.1).

Testing of rTbo-IT2 for the proton-activated hASIC1a and rASIC3 channels expressed in *Xenopus laevis* oocytes also showed no activity at concentrations up to 100 μM.

### 2.5. Spatial Structure of Tbo-IT2

The 3D structure of recombinant Tbo-IT2 was determined in a water solution by NMR spectroscopy. Based on torsion angle restraints, upper Nuclear Overhauser effect (NOE)-based distance restraints, disulfide and hydrogen restraints, 100 structures were calculated and the best 10 were selected for further analysis. Statistics of the input data and obtained structures is shown in Table 2. The set of structures is characterized by low CYANA target function, low backbone root-mean-square deviation (RMSD) value, and insignificant restraint violations, which indicates the high quality of the obtained structure.

**Figure 5:**
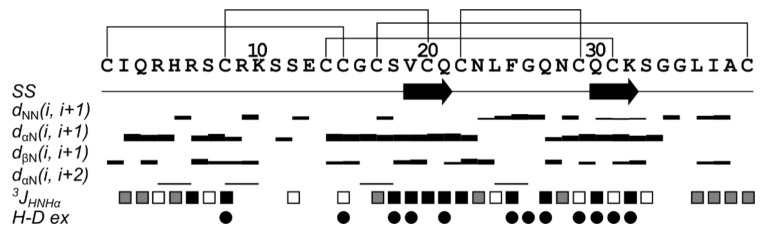
Overview of the NMR data that define the secondary structure of rTbo-IT2. Sequence, secondary structure (*SS*), NOE-connectivity, J-couplings, and hydrogen-deuterium exchange rate (*H-D ex*) are shown. Arrows indicate the β-strands of the β-sheet. Widths of the bars represent the relative intensity of cross-peaks in NOESY spectra. Colors of squares divide values of ^3^J_HNHα_ into three groups: low, <6 Hz (white); medium, 6–8 Hz (grey); large, >8 Hz (black). Circles denote the H_N_ protons, with the solvent exchange rates slower than 4 h^−1^.

**Figure 6:**
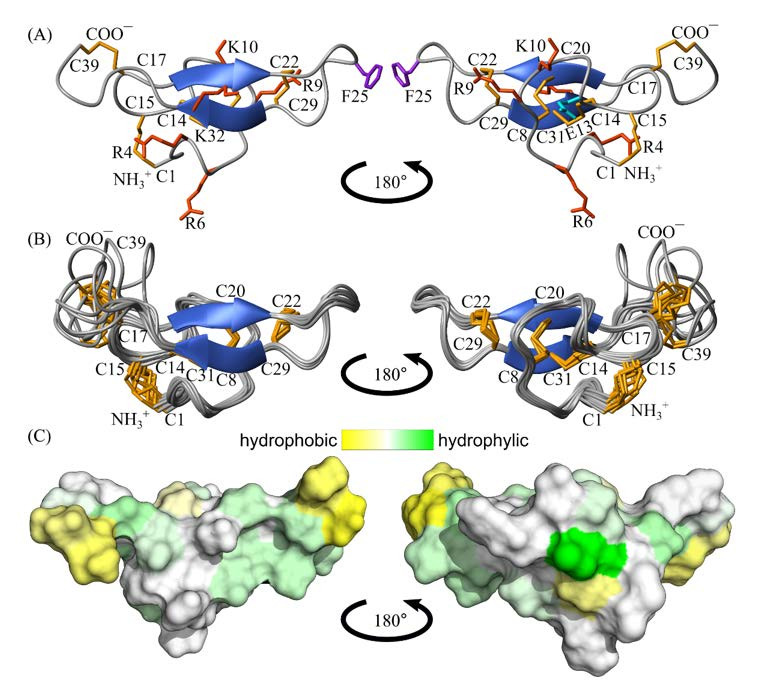
Two-sided view on the rTbo-IT2. (**A**) The structure with the fewest restraint violation. Disulfide bonds are colored in orange, positively charged amino acid residues are in red, negatively charged residues are in cyan, and aromatic residues are in purple. (**B**) The best 10 structures out of the initial 100 are superimposed on the backbone of β-sheet residues. Disulfide bonds are colored in orange. (**C**) The contact surface of rTbo-IT2 is colored according to the hydrophobicity, from yellow (hydrophobic) to green (hydrophilic) using the White and Wimley scale [24].

The 3D structure of rTbo-IT2 includes the 2-strand antiparallel β-sheet (strands V19-Q21 and Q30-K32), 7 β-turns (Q3-R6, IV type; K10-E13, I; S11-C14, IV; C15-S18, IV; N23-G26, I; G35-A38, I; L36-C39, VIII), and 3 γ-turns (Q3-H5, S12-C14, G16-S18). The structure is stabilized with 5 disulfide bonds (C1-C15, C8-C20, C14-C31, C17-39, C22-C29) and 9 hydrogen bonds. According to the obtained NMR data, the region of the last 6 amino acids (G35-C39) is flexible and unstructured. In contrast, the rest of the protein is stabilized and rigid. Overall, the surface of rTbo-IT2 is slightly hydrophilic, with small patches of hydrophobic residues (Figure 5 and Figure 6).

## 3. Discussion

It is known that animal venoms are the natural combinatorial libraries of biologically active molecules. Spider venom can contain up to several hundred molecules that are unique in their specificity and effectiveness. However, collecting a sufficient amount of venom, particularly for the small species, is difficult. In addition, individual components have rather low content in the venom. As a result, the structural and functional study of natural molecules is still a challenging task. The development of fast and sensitive transcriptome and proteome analysis methods, as well as efficient methods for recombinant peptide analogs production, significantly increased the capabilities for characterization of natural minor components of the venoms. In this work, we aimed to study one of the components of the *T. oblongus* spider venom, which was particularly interesting from the structural features point of view.

To investigate the diversity of polypeptide toxins encoded in mRNA of the *T. oblongus* spider venom glands, an expressed sequence tags (EST) library was constructed and sequenced. As a result, we obtained 1733 transcripts which were further analyzed. The mature toxins sequences were derived after the reading frame search and translation into proteins, followed by the isolation of signal and pro-peptides. Among the 217 molecules (unpublished data) that we classified as “toxins”, one of the peptides with an unusual motif of cysteine residues distribution was chosen for further study.

**Figure 7:**
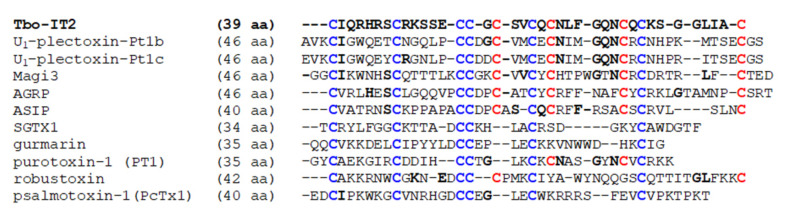
Sequence alignment of Tbo-IT2 and other inhibitor cystine knot (ICK) peptides. Cysteine residues contributed to the ICK fold are highlighted in blue, additional cysteine residues are in red.

The mature peptide Tbo-IT2 contains 10 cysteine residues. The BLASTp search for primary structure homologous molecules showed that Tbo-IT2 has very low amino acid sequence similarity with previously described molecules. The greatest similarity can be traced with 10-cysteine peptides from the group of plectoxins presented in the venom of *Plectreurys* spider genus [25,26] and the Magi3 (Mu-hexatoxin-Mg2a) peptide from the Hexathelidae spider *Macrothele gigas* [27] (~41% identical amino acid residues including 10 Cys residues) (Figure 7). At the same time, there is no complete coincidence in the number of amino acid residues between adjacent cysteine residues with any of these peptides.

As a result of proteomic analysis of the purified peptide fraction of the venom and its tryptic and GluC digests, it was unambiguously shown that the Tbo-IT2 peptide is present in the venom in a processed form, consisting of 39 amino acid residues and amidated at the C-terminus.

In order to have amounts of Tbo-IT2 needed for structural and functional studies, we used the heterologous gene expression method in a bacterial system, which we successfully used for a number of cysteine-rich natural molecules earlier [28,29,30]. The use of the fusion strategy with thioredoxin made it possible to obtain sufficient amounts of recombinant Tbo-IT2 with a high yield without additional refolding to determine the spatial structure of this peptide in solution.

NMR data showed that rTbo-IT2 is a well-structured compact molecule with a novel adaptation of ICK fold to 5 disulfide bonds configuration.

ICK fold is widely utilized by arachnids to fabricate a variety of toxins and it is believed to be an optimized and stable protein scaffold providing diverse biological activity [31]. Most common peptides forming ICK fold have 3 disulfide bridges (6 Cys residues) [4,31,32]. Some more complex molecules with 4 [13,32,33] and 5 disulfide bridges [27] also can form ICK-like fold. Plectoxins were found to be the closest primary structure homologs of Tbo-IT2 in the Swissprot database (Figure 7), but the closest molecules with reported spatial structure and very similar Cys distribution were Magi3 [27], agouti-signaling proteins (ASIP) [34], and agouti-related proteins (AGRP) [35].

**Figure 8:**
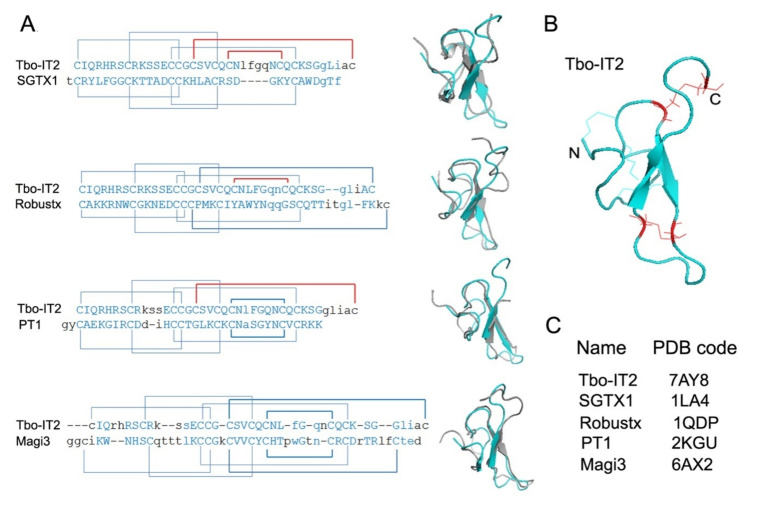
Comparison of Tbo-IT2 with other ICK peptides. (**A**) The primary structure alignment is based on spatial structure similarity with the SGTX1, robustoxin, purotoxin-1, and Magi3. The amino acid sequences are aligned according to the equivalent topological structure as determined by the PDBeFold algorithm. Residues that entered spatial alignment are shown in blue, whereas residues with a distance between Cα atoms more than 3 Å are shown in small letters. Similar disulfide bonds are shown in blue; additional disulfide bonds are shown in red (**B**) Spatial structure of Tbo-IT2, Cys17-Cys39, Cys22-Cys27 are shown in red; (**C**) Names and PDB codes of aligned peptides.

We performed an analysis of the spatial structure similarity using the PDBeFold analysis tool [36] and found that Tbo-IT2 could be aligned with the spatial structures of several peptides (Table 3 and Figure 8). The closest similarity of the spatial structure have ICK peptides: SGTX1 from the venom of the *Scodra griseipes* spider [37], gumarin isolated from the Indian plant *Gymnema sylvestre* [38], psalmotoxin-1 (PcTx1) from the venom of the South American tarantula *Psalmopoeus cambridgei* [39] with 6 Cys residues, robustoxin from the funnel-web spider *Atrax robustus* [40], and purotoxin-1 (PT1) from the venom of the wolf spider *Geolycosa* sp. [13] with 8 Cys residues. It is also noteworthy that the spatial structure of Tbo-IT2 is less similar to ASIP, AGRP, and Magi3 compared to other ICK peptides with 6 and 8 Cys residues. Therefore, the identical number of Cys and their similar distribution (Figure 8A) did not provide special features of 3D structure to 10 Cys ICK peptides.

The ICK motif in particular is characterized by the topology of the three disulfide bonds corresponding to C1-C15, C8-C20, and C14-C31 in Tbo-IT2 (Figure 7). Additional C17-C39 bridge in Tbo-IT2 most probably gives the C-terminal loop resistance to proteolytic degradation by carboxypeptidases (Figure 8B), similar features are observed for the creation of some 8 Cys ICK peptides such as robustoxin (Figure 8A). The disulfide bridge C22-C29 additionally stabilizes a protruding β-hairpin loop (Figure 8B), which also can be found in another type of Cys ICK such as purotoxin-1 [13] (Figure 8A) and Delta-palutoxin-IT2 [33]. We assume that 10 Cys peptides like Tbo-IT2, Magi3, and plectoxins employ these adjuncts found in different structures of 8 Cys ICK peptides and most probably benefit from both protective disulfide on the C-terminus and stable and extended central β-hairpin loop (Figure 8).

Earlier, we established a high insectoactivity of the whole venom of the spider *T. oblongus* [17]. Crude venom toxicity to *M. domestica* larvae was estimated as LD_50_ 10 μg/g for polypeptide fractions and several insectotoxic peptides were determined. The previously characterized 41-residue toxin ω-Tbo-IT1 demonstrated significant toxicity to insects. The activity of ω-Tbo-IT1 to *M. domestica* larvae was LD_50_ 19 μg/g [17]. Moreover, the action of ω-Tbo-IT1 on insect presynaptic Ca^2+^ channels was demonstrated, which allowed us to attribute the ω-Tbo-IT1 to the ω-group of toxins and inhibited voltage-gated calcium (Ca_V_) channels, according to the toxin’s classification [41]. The insectotoxicity of rTbo-IT2 (LD_100_ was 200 μg/g) is approximately 3 times lower than that one of ω-Tbo-IT1 (LD_100_ was about 65 μg/g [17], Figure 4), while there is no structural homology between these toxins.

The recombinant Tbo-IT2 and the natural toxin differ by the amidation of the C-terminal residue. Amidated peptides are common in venoms produced by various animals [42,43]. C-terminal amidation of peptides enhanced resilience to degradation by carboxypeptidases. Additionally, C-terminal amidation displays an important role in activity for some natural neurotoxins. Non-amidated toxins analogs in most cases showed activity several (from 4 to 15) times lower than the natural peptides [44,45,46,47,48] and sometimes C-terminal amidation was crucial for the activity of toxins [49,50,51]. Nevertheless, there are examples when amidated and non-amidated peptides exhibited almost the same activity [52,53,54], including spider toxins [14,55,56]. Apparently, amidation is a crucial factor when the overall charge of the molecule is important and/or the C-terminal residue is involved in receptor binding.

The isolation of native Tbo-IT2 toxin was impossible due to the insufficient amount of the venom. We admit that the activity of the recombinant Tbo-IT2, not amidated at the C-terminus, maybe slightly altered the activity of the native toxin. However, the rather high insectotoxicity of rTbo-IT2 suggests that peptide retained biological activity and the effect of C-terminal amidation is not so significant, and the recombinant analog can be used to search for the molecular target of Tbo-IT2.

The molecular target of Tbo-IT2 action is the most interesting matter. ICK toxins are active against sodium, potassium, calcium, TRP ion channels, ASICs, and ionotropic purinergic receptors [5,13,16,57]. ASIP and AGRP are endogenous antagonists of melanocortin receptors (MCR) that are seven transmembrane-domain proteins that couple to Gαs and the adenylate cyclase signal transduction pathway. ASIP normally controls pigmentation affecting the MC1R, while AgRP regulates body weight and metabolism and normally acts at the MC3R and MC4R [35].

It has been established that plectoxins can paralyze and/or kill insect pests such as *Heliothis. virescens* (lepidoptera), *Spodoptera exigua* (beet armyworm), and *Manduca sexta* (tobacco hornworm), while the molecular target of action has not been established [58].

Magi3 causes temporary paralysis to lepidopteran larvae (10.3 nmol/g) or crickets (doses from 0.93 to 119 µg/g) competing for binding at site 3 of the insect voltage-gated sodium channel and has no effects in mice at concentrations up to 20 pmol/g [27,59].

Close spatial structure homologs of Tbo-IT2 have a wide range of molecular targets.

SGTX1 (κ-theraphotoxin-Scg1a) inhibits outward K(+) currents in rat cerebellar granule neurons and weakly inhibits voltage-gated potassium channel Kv2.1 by shifting the channel activation to more depolarized potentials [37].

Robustoxin (δ-hexatoxin-Ar1a) is insecticidal and vertebrate-active toxin from one of the most dangerous spiders in the world—Sydney funnel-web spider *Atrax robustus*. Robustoxin is responsible for the symptoms observed in humans following envenomation by this spider, and robustoxin-neutralizing antidote has been used in Australia since 1981. Robustoxin slows the inactivation of both vertebrate tetrodotoxin-sensitive voltage-gated sodium (Nav) channels and insect para-type sodium channels by binding to site 3 of the channel [60]. A recent study showed that robustoxin induced pain in mice by inhibiting the inactivation of voltage-gated sodium channels involved in nociceptive signaling and that it also inhibited the inactivation of cockroach Nav channels and was insecticidal to sheep blowflies. It is not known whether it is specific for particular vertebrate Nav subtypes [61].

Purotoxin-1 was shown to exert notable modulatory effects on P2X3 receptors in rat sensory neurons [13]. Psalmotoxin-1 (π-theraphotoxin-Pc1a) has been reported to potently and specifically inhibit the homomeric ASIC1a channel [62]. Gurmarin is a highly specific sweet taste-suppressing protein in rodents, some of its amino acid residues had been identified as a putative binding site for the rat sweet taste receptor [38].

The most likely targets for Tbo-IT2 toxin are sodium or calcium channels. We tested the effect of recombinant Tbo-IT2 on the panel of available ion channels and neuroreceptors. However, we were unable to detect any activity in reasonable doses neither on insect (BgNav, DmNav, VdNav) and mammal (Nav1.4, Nav1.6, Nav1.7) sodium channels, nor various potassium channels (Shaker, Kv1.1, Kv1.2, Kv1.3, Kv1.4, Kv1.5, Kv1.6, Kv2.1, Kv3.1, Kv4.3, Kv10.1) and the calcium channel Cav3.1. rTbo-IT2 also had no effect on proton-activated hASIC1a and rASIC3 channels and TRP channels (rTRPV1, mTRPV2, hTRPV3, rTRPA1). To date, the Tbo-IT2 toxin target remains unknown.

## 4. Conclusions

rTbo-IT2 is a novel insecticidal toxin that adopts ICK fold with 5 disulfide bridges. Our results expand our understanding of the functional attributes brought about by structural variability observed in the ICK scaffold. The determined 3D structure of recombinant Tbo-IT2 showed significant similarity to ICK toxins with 3 and 4 disulfide bridges emphasizing the role of additional disulfide bridges in the stabilization of certain structural features (C-terminus and β hairpin) in sustainable ICK fold. This folding appears to make the peptide very target-oriented. rTbo-IT2 showed significant toxicity against *M. domestica* larvae. We were unable to identify the molecular target yet despite extensive screening effort, possibly because of a high selectivity exerted by this molecule.

## 5. Materials and Methods

### 5.1. Animal Handling

Crude *T. oblongus* venom was purchased from Fauna Laboratories, Ltd. (Almaty, Republic of Kazakhstan). Only female spiders were collected in the nearby Almaty region. Venom glands were extricated pairwise under Mustcam 1080P Full HD USB-microscope. Venom glands were dissected from several specimens and frozen in liquid nitrogen until sample preparation. To obtain a sufficient amount of mRNA from the venom glands, a preliminary (one week) milking procedure was performed to activate massive toxin expression. Crude venom for analysis was obtained by repetitive electrostimulation of 13 female spiders. Spiders were intensively fed by insects during intervals between electrostimulation.

### 5.2. EST Library Construction, Sequencing, and Data Processing

Total RNA from venom glands was extracted with SV Total RNA Isolation System (Promega, Madison, WI, USA). The yield and purity were assessed using a Nanodrop ND-1000 spectrophotometer (Thermo Fisher Scientific, Waltham, MA USA), while RNA integrity was determined by the RNA Integrity Number (RIN) using Bioanalyzer 2100 (Agilent Technologies, Santa Clara, CA, USA). The PCR-based cDNA library was created following the instructions for the SMART cDNA library construction kit (Clontech, Mountain View, CA, USA). Competent *E. coli* One ShotTOP10 cells (Thermo Fisher Scientific, Waltham, MA, USA) were transformed with cDNA library plasmids to amplify the cDNA. Plasmid DNA was purified with alkaline lysis and sequenced in 5′–3′ direction using ABI Prism 3730xl automatic DNA sequencer (Sanger technique) with BigDye Terminator version 3.1 cycle sequencing kit (Thermo Fisher Scientific, Waltham, MA, USA).

Basic operations with polynucleotide sequences such as open reading frames (ORF) detection, translation of the ORFs to proteins were performed using in-house scripts. Signal peptide coordinates were found using SignalP algorithm. Pro-peptide coordinated were detected using in-house scripts that take into account aspects of maturation of polypeptide spider toxins (so-called quadruplet motifs). For general data analysis and plot generation, both MS Excel and Origin 7.0 were exploited. All in-house scripts were either built-in Excel Visual Basic or short Python 3.0 scripts.

### 5.3. Venom Peptide Fraction Purification

1.6 mg of the whole venom were separated by reverse-phase HPLC (Jupiter C5 column 4.6 × 250 mm (5 μm; 300 Å), Phenomenex, Torrance, CA, USA) in a stepwise gradient of acetonitrile. Peptide fraction was eluted with 50% acetonitrile, evaporated under vacuum, and lyophilized twice. The obtained dry powder (1.3 mg) of venom peptide fraction had a light gray color and was used for proteomic analysis.

### 5.4. Peptide Digestion

Dried peptide fraction was dissolved in 50 mM ammonium bicarbonate buffer and reduced with 5 mM dithiothreitol (DTT) for 30 min at 60 °C. After that, iodoacetamide (IAA) was added to the samples up to the final concentration of 15 mM. The mixture was incubated in the dark at room temperature for 30 min. The resultant protein content (25–30 µg per sample) was digested with either trypsin or endoproteinase GluC (both enzymes from Promega, Madison, WI, USA) at the ratio of 1:75 w/w to the total protein content, and the mixture was incubated overnight at 37 °C for trypsin and at room temperature for GluC. After the reaction was stopped with 3% formic acid (final concentration), the sample was dried up using CentriVap micro IR Vacuum Concentrator (Labconco Corporation, Kansas City, MS, USA) at 45 °C. Dried peptides were stored at −45 °C until the LC-MS/MS analysis.

### 5.5. LC-MS/MS Analysis of Peptide Digests

The chromatographic separation of the digests was performed using Easy-nLC system (Thermo Fisher Scientific, Waltham, MA, USA). A home-made column (150 mm × 75 µm) with stationary phase Aeris™ 1.7 µm PEPTIDE XB-C18 100 Å (Phenomenex, Torrance, CA, USA) was used for the separation of peptide samples. A combination of mobile phase A, composed of 0.05% formic acid, 0.05% trifluoroacetic acid (TFA) in water, and mobile phase B, composed of 0.05% formic acid, 0.05% TFA, 10% water in acetonitrile (ACN), was used for analytical separation in the elution gradient at a flow rate of 0.3 µL/min. The following gradient was used for the separation of the digests: the concentration of mobile phase B had an increase from 0% to 60% over the first 60 min, then to 90% B over 10 min, followed by washing with 90% B for 20 min. The mass spectrometric analysis of the trypsin and GluC digests was performed on a high-resolution Orbitrap Elite mass spectrometer (Thermo Fisher Scientific, Waltham, MA, USA) in data-dependent acquisition mode with the following MS settings: scan resolution 60 K, maximum injection time 100 ms, automatic gain control 5 × 10^5^; and the following MS/MS parameters: 2 m/z isolation window width, top10 precursor selection method, dynamic exclusion time 10 s, normalized collision energy 28, fragmentation spectra resolution 30 K, maximum injection time 200 ms, automatic gain control 5 × 10^4^.

### 5.6. LC-MS Analysis of Intact Toxins

LC-MS analysis of intact toxin mixture was performed using high-resolution Orbitrap Velos mass spectrometer (Thermo Fisher Scientific, Waltham, MA, USA), coupled online with Agilent 1100 LC system with Zorbax 300 SB-C18 column (150 mm × 75 µm) (Agilent Technologies, Santa Clara, CA, USA). The solvents used for the peptide elution were water and ACN with 0.1% formic acid, the gradient included 10 min at 2% ACN for sample loading, followed by an increase to 5% ACN in 5 min, to 30% ACN in 100 min, and another step to 45% ACN in 15 min, followed by a wash at 95% ACN and re-equilibration. The mass spectrometer settings were as follows: scan resolution 100 K, maximum injection time 50 ms, automatic gain control 10^6^.

### 5.7. Proteomics Data Analysis

The protein sequence database in fasta format was built based on transcriptomic results with the addition of reversed sequences as decoys. The mass spectrometry raw data were converted to mgf format using Msconvert software [63]. The identifications were obtained using IdentiPy search engine [20]. The digest specificity was set to cleavage rules corresponding to the used proteases with a maximum of 4 missed cleavages allowed because of the low digestion efficiency of GluC endoproteinase. Carbamidomethylation of cysteine residues was set as a fixed modification, and due to the high concentration of alkylation agent, carbamidomethylation of methionine, histidine, lysine, and tryptophan residues was also included in the search as a variable modification, as well as C-terminal (protein-level) amidation. The precursor and fragment mass tolerances were set at 10 ppm and 0.02 Da, correspondingly. The post-search filtering to 1% false discovery rate (FDR) at peptide-spectrum match (PSM) level as well as data analysis, comparison, and visualization were performed using in-house Python language scripts based on Pyteomics library [20].

### 5.8. Gene Construction and Recombinant Analog Production

The DNA sequence encoding peptide Tbo-IT2 was constructed by PCR technique using five synthetic oligonucleotides: Tb-dir1 (AGA TAT CGA ATT CAA TGT GCA TTC AGC GTC ATC GTT CCT GC) containing the Met codon for BrCN cleavage, Tb-dir2 (CAG CGT CAT CGT TCC TGC CGT AAA TCC TCC GAA TGC TGC GGC TG), Tb-dir3 (CGA ATG CTG CGG CTG CTC CGT GTG CCA GTG CAA TCT GTT TGG), Tb-rev4 (CCG GAT TTG CAC TGG CAA TTC TGG CCA AAC AGA TTG CAC TGG), Tb-rev5 (CTC TCG AGT CAG CAC GCA ATC AGG CCG CCG GAT TTG CAC TG GC). The amplified PCR fragment was gel-purified and cloned into the expression vector pET32b+ (Merck, Darmstadt, Germany) digested with restriction enzymes EcoRI and XhoI (Thermo Fisher Scientific, Waltham, MA, USA). The assembled expression construct was sequenced and the complete correspondence of the cloned toxin’s DNA was confirmed. Recombinant peptide Tbo-IT2 was produced as a thioredoxin fusion protein in *E. coli* BL21(DE3). Competent *E. coli* BL21(DE3) cells were transformed with expression constructs and grown in Lysogeny broth (LB) culture medium containing 100 μg/mL ampicillin at 37 °C, moderate aeration and stirring to the optical density of OD_600_ ≈ 0.6–0.8. Next, to induce protein synthesis, isopropyl-1-thio-β-D-galactopyranoside (IPTG) was added to the culture medium to the final concentration of 0.2 mM, and the cells were incubated for 18 h at 25 °C. After cultivation, the cells were precipitated by centrifugation for 5 min at 6000 g, resuspended in a buffer for metal affinity chromatography (400 mM NaCl, 20 mm Tris-HCl, pH 7.5), and homogenized by ultrasound. The supernatant was centrifugated (15 min at 14,000 g) and the fusion protein was isolated from the supernatant using TALON Superflow metal affinity resin (Clontech, Mountain View, CA, USA) following the manufacturer’s recommendations. The cleavage of hybrid protein with BrCN was carried out using the previously developed method [23] in the dark, at room temperature, with the addition of HCl up to 0.2 M, and the BrCN molar ratio to protein was 600:1. The final peptides were purified by RP-HPLC on a Jupiter C5 column (300 Å, 10 μm, 250 × 10 mm) (Phenomenex, Torrance, CA, USA) in a linear gradient of ACN concentrations (0–20% over 2 min, 20–60% over 40 min) in the presence of 0.1% TFA with a constant flow rate of 5 mL/min. The purity of the recombinant peptide was confirmed by N-terminal sequencing and mass spectrometry.

### 5.9. Insectotoxicity Test

Insectotoxicity tests on *M. domestica* larvae were as described [17]. To estimate concentration-dependent effect, 5 doses were used (10, 25, 50, 100, 200 μg/g). The paralyzing effect development was monitored for 48 h. Each dose was measured on a group consisting of 12 larvae.

### 5.10. Activity Testing on Expressed Channels

All tests on expressed channels were performed according to previously described standard protocols. Testing for receptors rTRPV1, mTRPV2, hTRPV3, rTRPA1, stably expressed in CHO cells, was carried out using the calcium imaging method as described [64]. The maximal tested concentration of peptide was 1 μM. All other receptors were expressed in *Xenopus laevis* oocytes. Testing on hASIC1a and rASIC3 channels was according to the protocol [65], maximal tested concentration of peptide was 100 μM. Experiments on sodium (BgNav1.1, DmNav1, VdNav1, rNav1.4, mNav1.6, rNav1.7), potassium (Shaker, rKv1.1, rKv1.2, hKv1.3, rKv1.4, rKv1.5, rKv1.6, rKv2.1, hKv3.1, rKv4.3, hKv10.1), and calcium (rCav3.1) ion channels were made as described [66] with a maximal tested concentration of the peptide being equal to 5 μM.

### 5.11. NMR Spectroscopy and Calculation of Spatial Structure

All NMR experiments were run on the Avance III 600 MHz spectrometer (Bruker Biospin, Karlsruhe, Germany) at 30 °C. The protein was first dissolved in H_2_O/D_2_O (95:5), then in 100% D_2_O (CIL, Los Angeles, CA, USA). For the first case, pH was adjusted to 3.0, and TOCSY, NOESY (120 ms mixing time), ^1^H^13^C-HSQC, and ^1^H^15^N-HSQC were recorded. Based on these spectra ^1^H, ^13^C and ^15^N assignments were gained using the standard procedure [67]. In pure D_2_O, DQF-COSY and additional TOCSY, ^1^H^13^C-HSQC, and NOESY (100 ms mixing time) were recorded. A set of ^1^H spectra was also acquired in the first 30 min after dissolving the protein in D_2_O for measuring the rate of proton-deuterium exchange of amide groups.

All 3D structure calculations were performed in the CYANA software package version 3.98.13 using the simulated annealing/molecular dynamics protocol [68]. Torsion angles restraints and stereospecific assignment were obtained based on the J-couplings and NOE intensities. ^3^J_HNH__ɑ_ constants were determined from the analysis of NOE peaks line shape and ^3^J_H__ɑHβ_ constants were measured from the DQF-COSY spectrum using ACME [69]. Upper interproton restraints were obtained from r^−6^ calibration of NOESY cross-peaks. Disulfide bond connectivities were determined unambiguously in the course of the structure calculation. In the final stage of the calculations, the hydrogen bonds were introduced according to the proton–deuterium exchange rate. NMR chemical shifts and coordinates of rTbo-IT2 were deposited to the PDB database under the accession code 7AY8.

### 5.12. Computation

Homological polypeptides search was done using BLASTp tool in Uniprot Protein Knowledgebase (UniProtKB) and in ArachnoServer Spider Toxin database (http://www.arachnoserver.org). Offline computation, sequence alignments, and *E. coli* expression codon optimization were performed with Lasergene^®^ (DNAStar, Madison, WI, USA) programs.

## Figures and Tables

**Table 1:** Results of bottom-up proteomics identification of Tbo-IT2 toxin. Two unique (corresponding to one toxin of the database) tryptic peptides and one unique GluC peptide were identified in the corresponding searches with a significant number of peptide-spectrum matches (PSMs) per peptide. “AA-from” and “AA-to” columns show the position of the proteolytic peptide in the amino acid sequence of the toxin. GluC peptide covers the C-terminal region of Tbo-IT2, therefore, confirming its amidation.

Enzyme	PSMs	Peptide	AA-from	AA-to	Missed Cleavages
trypsin	11	KSSECCGCSVCQCNLFGQNCQCK	10	32	1
trypsin	8	SSECCGCSVCQCNLFGQNCQCK	11	32	0
GluC	12	CCGCSVCQCNLFGQNCQCKSGGLIAC-NH_2_	14	39	0

**Table 2:** Input data and validation statistics for the best 10 structures of rTbo-IT2.

Distance and Angle Restraints		
Total NOEs	361	
intraresidual	94	
interresidual	267	
sequential(|i − j| = 1)	78	
medium range(1 < |i − j| ≤ 4)	48	
long-range(|i − j| > 4)	141	
Hydrogen bond restraints(upper/lower)	23/23	
S-S bond restraints(upper/lower)	15/15	
J-couplings		
^3^J_HNHα_	29	
^3^J_HαHβ_	42	
Angles		
φ	38	
Χ_1_	18	
**Total restraints/per residue**	484/12	
**Statistics of the obtained set of structures**		
CYANA target function	1.64 ± 0.25	
Restraints violations		
distance(>0.2 Å)	0	
angle(>5°)	2	
RMSD(Å)	SS ^1^	RR ^2^
backbone	0.18 ± 0.03	0.57 ± 0.11
all heavy atoms	0.76 ± 0.07	1.40 ± 0.17
Ramachandran analysis *		
%residues in most favored regions	69.7	
%residues in additional allowed regions	30.3	
%residues in generously allowed regions	0.0	
%residues in disallowed regions	0.0	

^1^ SS—secondary structure, ^2^ RR—rigid region. * Ramachandran analysis was performed with PROCHECK tool on RCSB validation server (deposit.rcsb.org/validate/).

**Table 3:** Results of PDBeFold structure alignment for Tbo-IT2 (7AY8). % ident—fraction of pairs of identical residues among all aligned. The Q-score is calculated as Q = Nalgn × Nalgn/((1 + (RMSD/R_0_)^2^) × Nres1 × Nres2), where R_0_ = 3 Å. The Z-score is the measure of the statistical significance of a match. The higher the Z-score, the higher the statistical significance of the match.

Peptides	PDB Codes	N Res	N Align	% Ident	Scoring		RMSD
					Q	Z	
SGTX1	1la4:A	34	33	21	0.63	6.2	1.68
robustoxin	1qdp:A	42	38	26	0.60	5.8	2.06
gurmarin ^1^	5oll:A	35	30	20	0.54	4.6	1.43
PT1	2kgu:A	35	31	35	0.53	6.3	1.72
PcTx1	1lmm:A	40	33	21	0.52	4.1	1.77
ASIP(80-132:Q115Y, S124Y)	1y7j:A	40	34	29	0.43	3.6	2.52
AGRP(87-132)	1hyk:A	46	33	36	0.43	4.6	1.92
Magi3	6ax2:A	46	31	32	0.34	3.6	2.24

^1^ Crystal structure.

## Data Availability

The data presented in this study are openly available in PDB database under the accession code 7AY8.

## Supplementary Materials

The following are available online at https://www.mdpi.com/2072-6651/13/1/29/s1, Figure S1: RP-HPLC isolation of recombinant Tbo-IT2: (A) separation of BrCN cleavaged hybrid protein on a Jupiter C5 column (300 Å, 10 μm, 250 × 10 mm) (Phenomenex, Torrance, CA, USA) in a linear gradient of ACN concentrations (0–20% over 2 min, 20–40% over 20 min, at 18 min, switching the gradient to 80% over 5 min) in the presence of 0.1% TFA with a constant flow rate of 5 mL/min; (B) final rTbo-IT2 purification on the same column in a linear gradient of ACN concentrations (0–20% over 2 min, 20–60% over 40 min) in the presence of 0.1% TFA with a constant flow rate of 5 mL/min., Figure S2: The mass-spectrum of recombinant Tbo-IT2. The molecular weight of rTbo-IT2 was measured on a high-resolution Orbitrap Elite mass spectrometer (Thermo Fisher Scientific, Waltham, MA, USA) by direct sample injection. Mass difference between calculated and measured rTbo-IT2 +4H^+^ is 0.03 Da. Measured rTbo-IT2 monoisotopic mass (4201.56 Da) corresponds to theoretically calculated one (4201.69 Da) with five disulfide bonds and without C-terminal amide.
