# Supplementary material for: New Insectotoxin from Tibellus Oblongus Spider Venom Presents Novel Adaptation of ICK Fold
Source: Toxins (Basel). 2021 Jan 4;13(1):29. doi: 10.3390/toxins13010029 (PMC7824768; doi:10.3390/toxins13010029)
Supplement: Supplementary file 1 [file toxins-13-00029-s001.pdf]

Supplementary Materials: New Insectotoxin from Tibellus Oblongus Spider Venom Presents Novel Adaptation of ICK Fold

Yuliya Korolkova, Ekaterina Maleeva, Alexander Mikov, Anna Lobas, Elizaveta Solovyeva, Mikhail Gorshkov, Yaroslav Andreev, Steve Peigneur, Jan Tytgat, Fedor Kornilov, Vladislav Lushpa, Konstantin Mineev and Sergey Kozlov

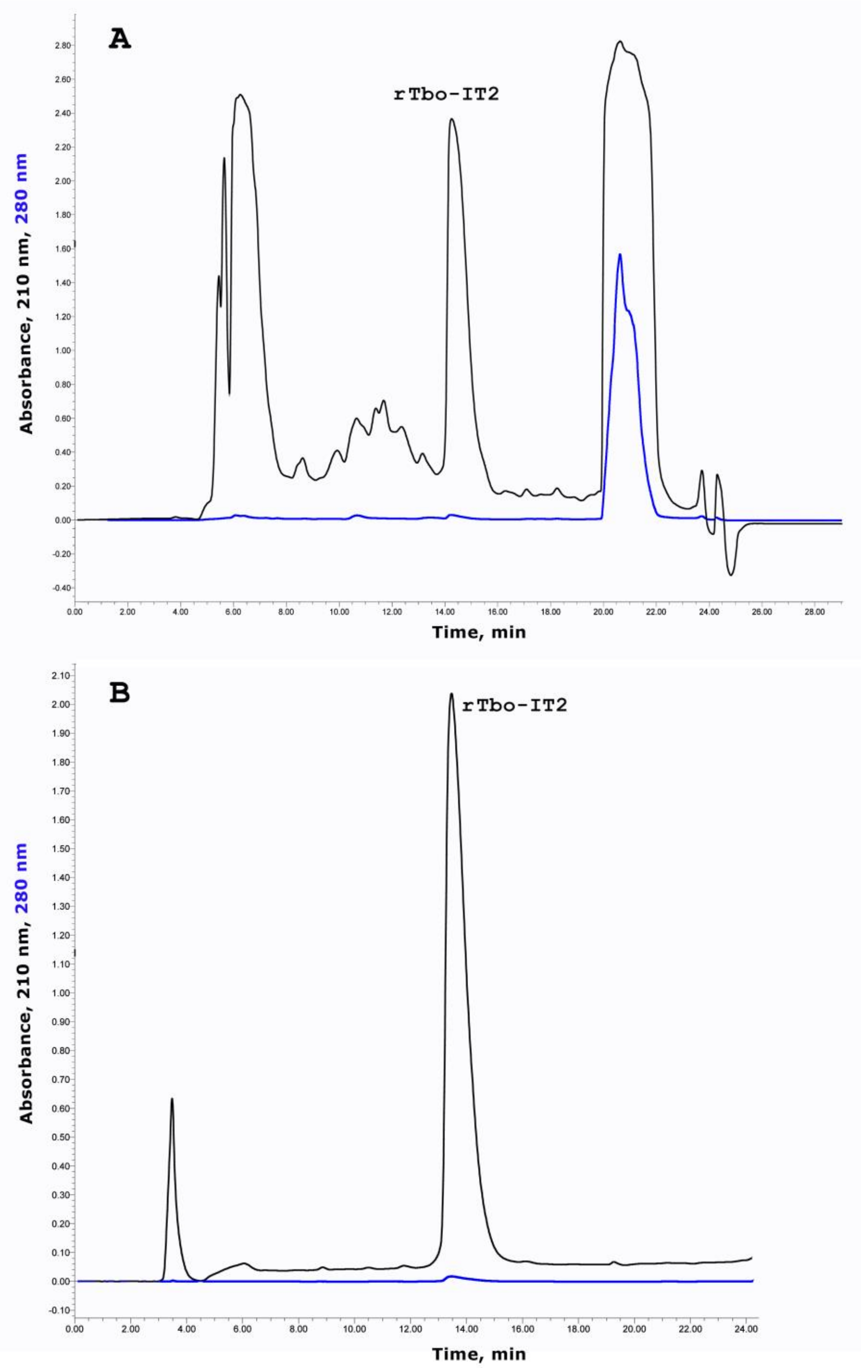

Figure S1. RP-HPLC isolation of recombinant Tbo-IT2: (**A**) separation of BrCN cleavaged hybrid protein on a Jupiter C5 column (300 Å, 10 µm, 250 × 10 mm) (Phenomenex, Torrance, CA, USA) in a linear gradient of ACN concentrations (0–20% over 2 min, 20–40% over 20 min, at 18 min, switching the gradient to 80% over 5 min) in the presence of 0.1% TFA with a constant flow rate of 5 mL/min; (**B**) final rTbo-IT2 purification on the same column in a linear gradient of ACN concen-

trations (0–20% over 2 min, 20–60% over 40 min) in the presence of 0.1% TFA with a constant flow rate of 5 mL/min.

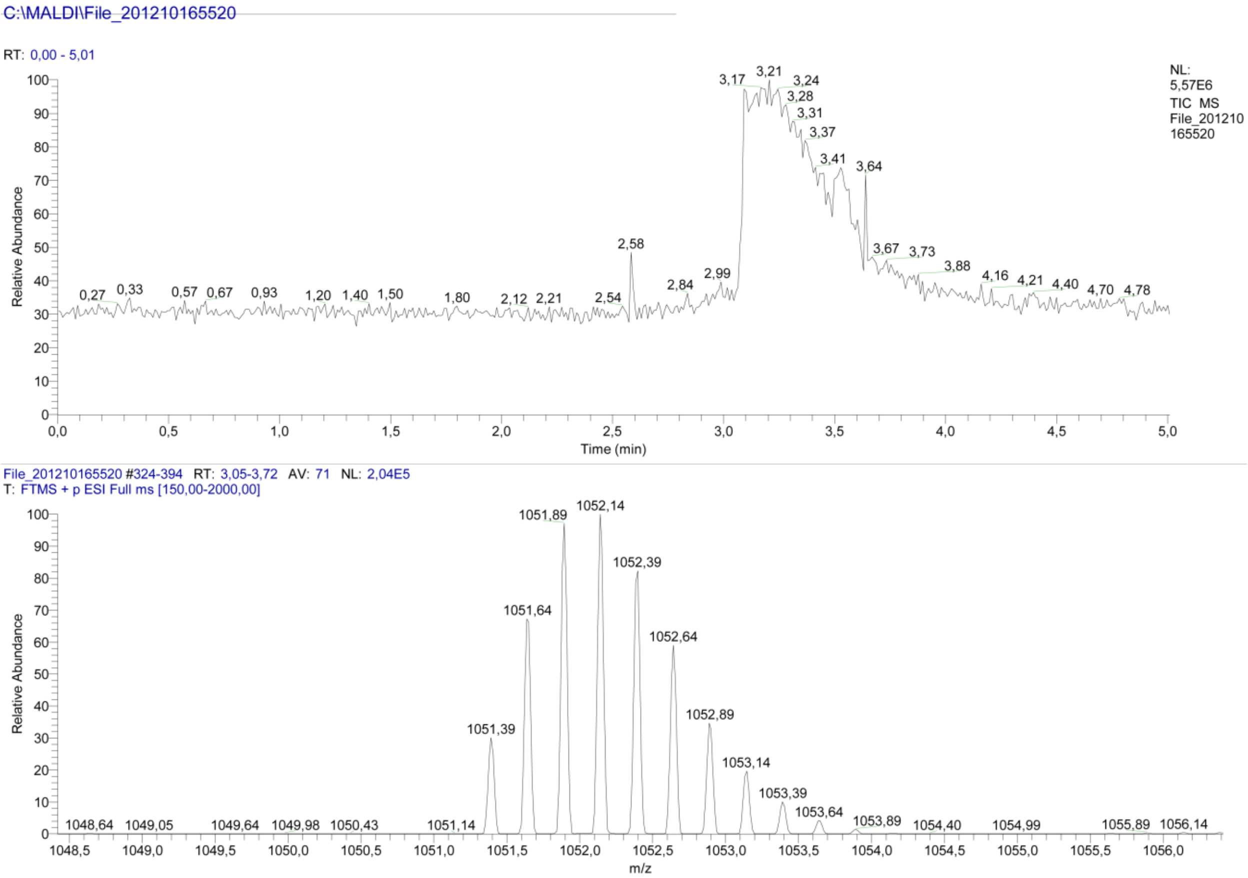

Figure S2. The mass-spectrum of recombinant Tbo-IT2. The molecular weight of rTbo-IT2 was measured on a high-resolution Orbitrap Elite mass spectrometer (Thermo Fisher Scientific, Waltham, MA, USA) by direct sample injection. Mass difference between calculated and measured rTbo-IT2 +4H$^+$ is 0.03 Da. Measured rTbo-IT2 monoisotopic mass (4201.56 Da) corresponds to theoretically calculated one (4201.69 Da) with five disulfide bonds and without C-terminal amide.
